# A Single-Indicator Factor Approach for Correcting Measurement Error in Time-Varying Predictors in Developmental Research

**DOI:** 10.3390/bs16060855

**Published:** 2026-05-27

**Authors:** Kejin Lee

**Affiliations:** Department of Education, Pusan National University, Faculty Office Building 2-#403, 2, Busandaehag-ro, 63Beon-gil, Geumjeong-gu, Busan 46241, Republic of Korea; kejin@pusan.ac.kr

**Keywords:** simulation study, latent growth modeling, single-indicator factor

## Abstract

Composite scores (e.g., mean or sum of survey items) are widely used as outcomes or predictors in psychological and social science research despite methodological concerns regarding measurement error. Despite extensive study of measurement error in path models, relatively little attention has been paid to this methodological issue in latent growth modeling (LGM), particularly when predictors vary over time. Time-varying predictors allow for modeling occasion-specific influences beyond underlying developmental trajectories, yet they are frequently operationalized using composite scores that implicitly assume perfect reliability. To address this gap, the present study investigates the consequences of ignoring measurement error in composite time-varying predictors within the LGM framework. Notably, this is the first study to evaluate the single-indicator (SI) modeling approach as a method for correcting measurement error in time-varying predictors. We compared the traditional LGM incorporating composite predictors with the LGM that incorporates the SI factor approach to account for measurement error in time-varying predictors using the Early Childhood Longitudinal Study, Kindergarten Class of 1998–1999 (ECLS-K) dataset and a Monte Carlo simulation. The Monte Carlo simulation results revealed that ignoring measurement error in time-varying predictors attenuated occasion-specific effects by up to 30%. These findings underscore the necessity of correcting for measurement error using SI factor modeling to ensure the validity of developmental inferences in LGM.

## 1. Introduction

Use of composite scores as outcomes or predictors in psychological and educational research has been widely discussed alongside growing efforts to evaluate and improve methods for correcting potential measurement error over the past decades (Cole & Preacher, 2014; Hsiao et al., 2018; Lai et al., 2023). In practice, psychological and social science researchers have frequently adopted composite scores (e.g., mean scores or sums of survey items) as outcomes or predictors when measurement invariance assumptions for the variables of interest are not met (Hsiao et al., 2018, 2021). In more recent years, cautionary discussions regarding the use of predictors without correcting for measurement errors have become increasingly discussed in psychological research (e.g., Cole & Preacher, 2014; Hsiao et al., 2018, 2021; Lai et al., 2023).

More specifically, Hsiao et al. (2018) reviewed articles published in *Journal of Applied Psychology* in 2015 (*N* = 120) and reported that applied researchers predominantly relied on composite scores (e.g., means or sums of multiple items). Their review revealed that approximately 90% of the articles used observed composite scores, with only a small proportion correcting for potential measurement errors. These findings are consistent with Cole and Preacher’s (2014) findings that approximately 12% of studies conducting path analysis failed to correct for measurement error in outcomes or predictors after reviewing American Psychological Association journals. According to Hsiao et al. (2018), ignoring measurement errors in predictor variables and their associated interaction effects is frequently found in educational and psychological research. When applied researchers use manifest variables as predictors without correcting for measurement error, biased path coefficient estimates are likely to result because perfectly reliable manifest variables are rarely observed in real-world data (Cole & Preacher, 2014; Hsiao et al., 2018; Konold & Sanders, 2025; Lai et al., 2023).

While prior simulation studies investigated the effects of ignoring measurement error in path analyses or predictor variables (e.g., Hsiao et al., 2021), the impact of measurement error in time-varying predictors has received little attention and the literature addressing time-varying predictors remains limited. In latent growth modeling (LGM), time-varying predictors enable the investigation of occasion-specific variability. Therefore, this study examines the methodological advantages of modeling time-varying predictors as single-indicator latent factors in the estimation of occasion-specific effects, given their importance in psychopathological and developmental research contexts. Within the LGM framework, repeated outcome measures are modeled as functions of an underlying latent growth trajectory and temporal fluctuations in predictors across measurement occasions (Curran et al., 2010; Curran & Hussong, 2003). This approach is ideally suited for developmental research, where short-term shifts in contextual or psychological factors are theorized to exert meaningful influence beyond long-term trends.

In practice, composite scores are commonly used for time-varying predictors in LGM. When doing so, the item measures of each composite score are assumed to be psychometrically perfectly reliable, which is uncommon. This can be problematic in that the use of composite scores ignores potential measurement error in the scale items (Leite, 2007) and the path coefficient associated with the composite scores can be attenuated due to this unreliability (Cole & Preacher, 2014). Importantly, Leite (2007) examined ways to correct for measurement error in composite outcomes within the LGM framework and introduced a single-indicator (SI) factor approach (Bollen, 1989; Hayduk, 1987) to handle the unreliability in the composite outcomes in LGM.

Although Leite (2007) applied the SI factor approach to composite outcome variables in LGM, resulting in endogenous SI factors, no study to date has examined the impact of modeling measurement error in composite time-varying predictors using this approach. When measurement error in time-varying covariates is modeled, the resulting SI factors are exogenous. However, no study has examined the use of SI factors with time-varying predictors. Therefore, the primary aim of this study is to address this methodological gap by evaluating the consequences of ignoring measurement error in time-varying predictors using the SI factor approach. To achieve this, this study first compared traditional composite-based LGM with the SI factor model using the Early Childhood Longitudinal Study, Kindergarten Class of 1998–1999 (ECLS-K) dataset to observe real-world parameter discrepancies. In addition, a Monte Carlo simulation study was conducted to evaluate parameter bias and standard error recovery under conditions in which measurement error in time-varying predictors is either ignored or appropriately modeled.

## 2. Materials and Methods

### 2.1. Latent Growth Modeling with Time-Varying Predictors

Latent growth modeling (LGM, or latent curve model; Meredith & Tisak, 1990) in structural equation modeling (SEM) specifies a growth trajectory with outcomes measured across time as a function of two latent growth factors that are labeled as intercept and slope factors. Specifically, the intercept factor represents the initial status of an outcome variable when using the first measurement occasion as the reference point, and the slope factor indicates the change in growth over time (Bollen & Curran, 2006).

The LGM framework can be extended to allow for including a time-varying predictor measured at each measurement occasion. By incorporating the time-varying predictor, the repeatedly measured outcome variables are explained by a latent growth trajectory and the occasion-specific effects of the time-varying predictor (Curran et al., 2010; Curran & Willoughby, 2003). The occasion-specific effects of the time-varying predictor are often primary effects of interest in psychopathological research. As such, conditional LGM with a time-varying predictor has frequently been used for examining developmental theories (Brammer et al., 2016; Curran & Hussong, 2003). Because the composite scale scores (e.g., mean and sum of multiple survey items) are commonly used as repeatedly measured time-varying predictors in the conditional LGM framework, the occasion-specific effects of the time-varying predictor on its corresponding outcome measure could be biased due to the unreliability in its measurement (Cole & Preacher, 2014). In such cases, the unreliability of the composite time-varying predictor may be corrected by implementing the SI factor approach (Bollen, 1989; DeShon, 1998; Hayduk, 1987).

While Leite (2007) applied the SI factor approach to the LGM framework, he explained the equivalence of the parameter and standard error estimates from the traditional, unconditional LGM and those from an unconditional LGM in which the latent growth trajectory is measured by multiple SI latent variables due to the endogeneity of the SI factors. However, measurement error can be handled using an SI factor approach that is incorporated into each time-varying predictor in conditional LGM in which the predictor is exogenous. To our knowledge, no study has explored the impact of correcting for measurement errors in time-varying predictors in LGM.

Correspondingly, the purpose of this study is to examine the effects of ignoring measurement error in conditional LGM with a time-varying predictor. First, this study demonstrated differences that can result from ignoring measurement error in composite scores used as time-varying predictors with use of the SI factor for handling that measurement error via the Early Childhood Longitudinal Study, Kindergarten Class of 1998–1999 (ECLS-K) dataset. Second, we also conducted a simulation study to assess parameter and standard error recovery for the models that ignore versus correct for measurement error in time-varying predictors. In the simulation study, we tested estimation of the models under conditions in which we manipulated sample size, the number of measurement occasions, the degree of reliability in the time-varying predictor, and the magnitude of the occasion-specific coefficient for the time-varying predictor.

### 2.2. Consequences of Ignoring Measurement Error

Combining educational or psychological scale items into scale scores or composite scores (e.g., mean or sum of survey items) is frequently seen in practice and the composite scores are commonly used as outcomes in traditional LGM. When doing so, the item measures of each composite score are assumed to be psychometrically reliable. However, this can be problematic in that the use of composite scores ignores the potential measurement errors in the multiple indicators or items (Leite, 2007), and traditional path analysis modeling techniques do not provide a capability for partitioning out measurement errors in the composite score from the model (Stephenson & Holbert, 2003). The measurement errors generally refer to the degree of random (i.e., unsystematic) errors that affect the accuracy of the measurement for a given variable.

**Figure 1:**
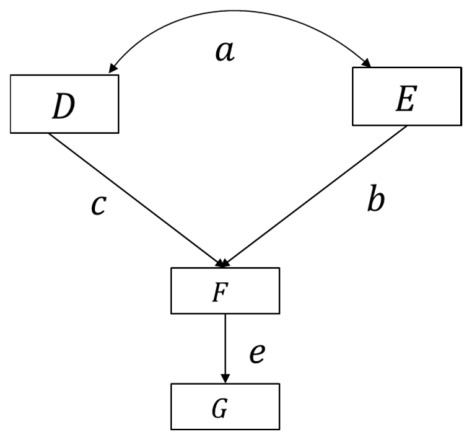
Cole and Preacher’s (2014) example of a “Fallible Triangle.”.

Importantly, Cole and Preacher (2014) demonstrated the consequences of ignoring measurement errors in any of the variables in path analyses. The authors highlighted how directional path coefficients can be underestimated or overestimated when there is potential unreliability in any of the variables in a path analysis model. Specifically, unreliability in any single variable in a path analysis model introduce a “pressure” either to inflate or to attenuate certain path coefficients in the model. For instance, in Figure 1, measurement errors in variable *D* and variable *F* show pressures to attenuate a path ***c***, whereas measurement errors in variable *E* introduce pressure to inflate path ***c***. That is, a directional path coefficient can only be underestimated with uncorrected measurement errors in the two variables that are related to the path (Cole & Preacher, 2014). Likewise, the magnitude of the path coefficient from a variable *F* to a variable *G* in Figure 1 (i.e., path *e*) is attenuated by the measurement errors in either *F* or *G*. Moreover, the measurement errors in *F* and *G* can affect the path coefficient even when the path coefficient “*e*” is constrained to zero because the two variables’ pressures on the path “*e*” are not eliminated (Cole & Preacher, 2014). By conducting a Monte Carlo simulation study, Cole and Preacher (2014) found that measurement error in any variable always attenuated the path coefficients that are involved with the variable. Additionally, in a situation where the true path coefficient was zero, the measurement errors in the variables connected to the path inflated those path coefficients.

One way to handle the measurement errors in the observed scale scores or composite scores in LGM is the curve-of-factors model (McArdle, 1988), which is also called a second-order model (Hancock et al., 2001). The curve-of-factors model is a function of a common measurement model and a latent growth model, while the latent growth trajectory is based on the repeated latent variables that are represented by multiple scale items (indicators). The measurement part of the curve-of-factors model determines the extent to which the multiple indicators assess their corresponding latent variables at each time point. The latent growth part determines the aspects of growth trajectories via the intercept and slope factors of the latent variables across time. However, the curve-of-factors model has not been widely used in longitudinal studies because it requires a relatively large sample (e.g., *N* = 500) and researchers are unlikely to have information about item-level data in many cases (Leite, 2007).

An alternative way to control for the possible measurement error in composite scores in a path analysis involves use of the SI approach. That is, the error variance of a single composite score is constrained by an estimate of the reliability of the composite score, and a latent variable (SI factor) that is represented by the single composite score can now be used that represents the true, perfectly reliable score underlying the composite score (Bollen, 1989; DeShon, 1998). Although Cronbach’s α is widely used as an index of internal consistency, it accurately reflects reliability only under essential tau-equivalence, which is an assumption that is rarely found in applied research (Cho, 2024a). When items are congeneric, Cronbach’s α tends to underestimate true reliability, a limitation that has been well-documented in prior simulation studies (e.g., Cho, 2024a, 2024b), which may in turn lead to overestimation of the adjusted parameter values. However, since this issue falls outside the primary scope of the present study, the implications of tau-equivalence violations are not examined further here. The path between the observed composite score and the unique factor is typically set to 1.0 for model identification purposes (DeShon, 1998). An SI factor can be represented as follows:

(1) $$y_{i}=\lambda_{t}\eta_{i}+\varepsilon_{i},$$

where $y_{i}$ represents a single composite score for the *i*th individual, $\lambda_{y}$ is a factor loading that loads on the SI factor ($\eta_{i}$) at time point *t*, $\eta_{i}$ is a latent variable for the *i*th individual, and $\varepsilon_{i}$ are residuals in outcome measure that are not explained by the factor ($\eta_{i}$) for the *i*th individual. The error variance in $y_{i}$ can be expressed as follows:

(2) $$\sigma_{\varepsilon_{i}}^{2}=(1-\rho_{yy\prime })\sigma_{y_{i}}^{2},$$

where $\sigma_{\varepsilon_{i}}^{2}$ is the error variance of the composite score ($y_{i}$) for the *i*th individual, $\rho_{yy\prime }$ is the internal consistency reliability (e.g., Cronbach’s alpha) of the composite score ($y_{i}$), and $\sigma_{y_{i}}^{2}$ is the estimated variance of the composite score. By constraining the error variance of the composite score to equal [(1 − $\rho_{yy\prime }$)$\;\sigma_{y_{i}}^{2}$] and setting the factor loading ($\lambda_{y}$) to 1.0, the SI measurement model can be estimated.

Consequently, the measurement error of the composite score can be accounted for by specifying a latent factor on which the composite score loads as a single indicator; that is, a single composite score represents an indicator of the SI factor ($\eta_{ij}$) and the error variance of the composite score is fixed to a constant capturing the measurement error. Once the error variance of the composite score is fixed, the disturbance variance of the factor (i.e., reliable variance of the composite score) is freely estimated and the factor (i.e., the error-free true score for the composite score) can be used in a model. The benefits of using a single-indicator factor rather than a composite score include that it can improve the accuracy of parameter and standard error estimates and enhance the power to detect true effects of interest (Cole & Preacher, 2014; Stephenson & Holbert, 2003).

### 2.3. Conditional LGM with a Single-Indicator Time-Varying Predictor

**Figure 2:**
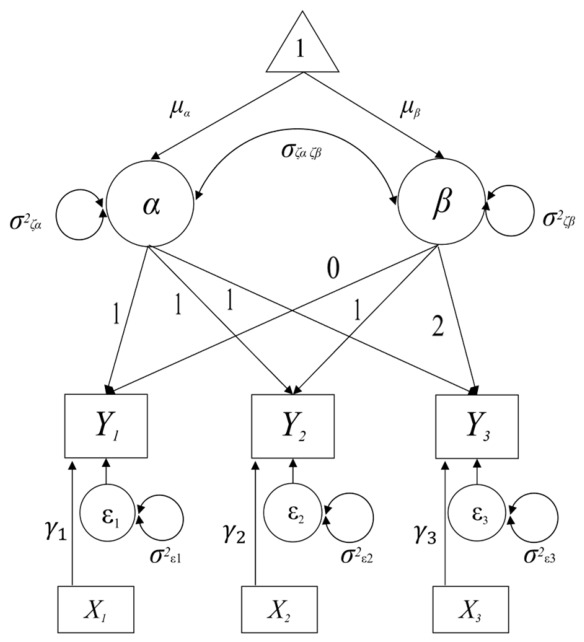
Conditional LGM with a composite time-varying predictor.

**Figure 3:**
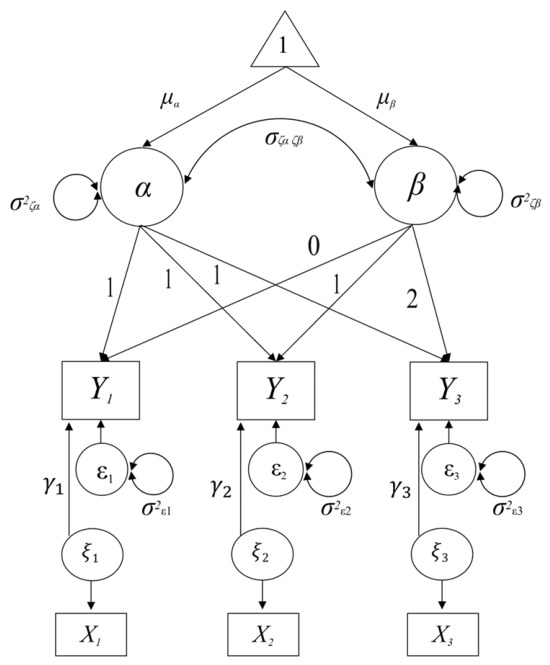
Conditional LGM with a single-indicator factor time-varying predictor.

A conventional conditional LGM with a time-varying predictor is represented in Figure 2 and the model can be extended to include the SI factor measurement model, as seen in Figure 3.

That is, a factor ($\xi_{it}$) that is represented by a single indicator (composite score), or an observed time-varying predictor (*x_it_*) for time point *t* for the *i*th individual, can be incorporated into the conditional LGM. The SI measurement model in Figure 3 can be expressed as follows:

(3) $$x_{it}=\;\xi_{it}+\epsilon_{it},$$

where $x_{it}$ is a time-varying predictor (a composite score) measured at time point *t* for the *i*th individual in Equation (3), $\xi_{it}$ represents an SI factor that is represented by a single indicator ($x_{it}$) for the *i*th individual at time point *t* with a factor loading constrained to 1.0, and $\epsilon_{it}$ is the error portion of the variance in $x_{it}$ that is not explained by its corresponding SI factor ($\xi_{it}$). Accordingly, the reliable portion or true score of the occasion-specific predictor $x_{it}$ is represented by its corresponding unique factor ($\xi_{it}$). Subsequently, the conditional LGM with the SI measurement model as seen in Figure 3 can be represented as follows:

(4) $$y_{it}=\;\alpha_{i}+\beta_{i}\lambda_{t}+\gamma_{t}(\xi_{it}+\epsilon_{it})+\varepsilon_{it},$$

where $y_{it}$ is the observed outcome for individual *i* at time point *t*; $\alpha_{i}$ is the latent intercept parameter for the *i*th individual. $\beta_{i}\lambda_{t}$ is the latent slope parameter that is multiplied by factor loadings representing a measured time scale. For the SI measurement model, $\xi_{it}$ indicates an SI factor that is represented by a single indicator ($x_{it}$) for the *i*th individual at time point *t* with a factor loading constrained to 1.0, and $\epsilon_{it}$ is the error portion of the variance in $x_{it}$ that is not explained by its corresponding SI factor ($\xi_{it}$). Accordingly, $\gamma_{t}\xi_{it}$ is the occasion-specific effect of the SI factor ($\xi_{it}$) that is represented by its corresponding time-varying covariate $x_{it}$ for the *i*th individual at time point *t*, since $\gamma_{t}$ signifies the unique effect of the predictor directly upon the occasion-specific measure of the outcome. ${\gamma_{t}\epsilon }_{it}$ is the error portion of the variance in $x_{it}$ that is not explained by its corresponding SI factor ($\xi_{it}$) and $\varepsilon_{it}$ is the measurement error for the *i*th individual at time point *t*. The measurement errors are assumed to be uncorrelated with the time-varying predictor at each time point (Preacher et al., 2008). Specifically, the outcome variable is now a joint function of the random coefficients plus the time-specific influences associated with the time-varying SI factor or exogenous variable ($\xi_{it}$). The matrix format of Equation (4) is represented as follows:

(5) $$\left[\begin{array}{c} y_{i1}\\ y_{i2}\\ y_{i3} \end{array}\right]=\;\left[\begin{array}{cc} 1 & 0\\ 1 & 1\\ 1 & 2 \end{array}\right]\left[\begin{array}{c} \alpha_{i}\\ \beta_{i} \end{array}\right]+\left[\begin{array}{ccc} \gamma_{i1} & 0 & 0\\ 0 & \gamma_{i2} & 0\\ 0 & 0 & \gamma_{i3} \end{array}\right]\left[\begin{array}{c} \xi_{i1}+\epsilon_{i1}\\ \xi_{i2}+\epsilon_{i2}\\ \xi_{i3}+\epsilon_{i3} \end{array}\right]+\left[\begin{array}{c} \varepsilon_{i1}\\ \varepsilon_{i2}\\ \varepsilon_{i3} \end{array}\right].$$

Consequently, the occasion-specific predictor (*x_it_*) in Equation (3) is now replaced by the measurement-error-free SI factors ($\xi_{it}$), as seen in Equation (4). In other words, the measurement error in the observed occasion-specific predictor (*x_it_*) is now controlled and thereby the magnitude of the time-specific effect (*γ_t_*) is not attenuated by measurement error in the predictor.

### 2.4. Real Data Analysis

#### 2.4.1. Empirical Dataset

Data were retrieved from the Early Childhood Longitudinal Study, Kindergarten Class of 1998–1999 (ECLS-K). The ECLS-K is a widely used and nationally representative sample of U.S. children. More specifically, the data were collected in the fall and the spring semesters of the children’s kindergarten year (1998–1999), which is the baseline year. Then, data were collected in the fall and spring semesters of first grade (1999–2000), the spring semester of third grade (2002), the spring semester of fifth grade (2004), and the spring semester of eighth grade (2007). From the ECLS-K original sample (*N* = 21,409), the example data that were used for this current study included students who were measured on their mathematics ability at five time points: the fall semester of kindergarten, the spring semester of kindergarten, the spring semester of first grade, the spring semester of third grade, and the spring semester of fifth grade. A subsample based on the fall semester of first grade measurement occasion was excluded in this study because the fall of first grade sample only encompasses a 27% subsample of the total sample. Further, those students who changed schools between the fall of kindergarten and the spring of fifth grade were also excluded. Accordingly, a total of 200 students were randomly selected from the sample in which students did not change school between the fall of kindergarten and the spring of fifth grade, excluding incomplete responses in the mathematics theta score variables, as the final sample for this example data analysis. To eliminate potential clustering effects at the school level, the sample was based on 200 selected students who were each drawn from a different school. Therefore, no nesting structure was present in the final dataset.

#### 2.4.2. Variables

The variables that were used in this study included students’ mathematics theta composite scores (i.e., C1R4MTHT, C2R4MTHT, C4R4MTHT, C5R4MTHT, C6R4MTHT), reading theta composite scores (i.e., C1R4RTHT, C2R4RTHT, C4R4RTHT, C5R4RTHT, C6R4RTHT), and the reliabilities of reading theta composite scores. Specifically, the students’ mathematics theta scores represent their underlying mathematical ability and the students’ reading theta scores represent their underlying reading ability, ranging from −3 to 3. The theta scores are generally known to be well-suited for assessing students’ growth. Further, the reading theta scores are mean-centered so that students who scored zero in the time-varying predictor (reading score) represent the students at the average level in reading ability. Additionally, the reliabilities that are used for reading theta scores in this study are 0.92 (the fall of kindergarten), 0.95 (the spring of kindergarten), 0.96 (the spring of first grade), 0.94 (the spring of third grade), and 0.93 (the spring of fifth grade) (Tourangeau et al., 2009).

#### 2.4.3. Statistical Analysis

The data were analyzed using the M*plus* (ver. 7.4, L. Muthén & Muthén, 2012) software program. The conditional LGM without the SI approach (LGM composite model; see Figure 2), and the conditional LGM with the SI approach (LGM SI model; see Figure 3) were fitted to the given (final) ECLS-K sample that is described in previous sections. Missing data in the empirical data analyses were handled using full information maximum likelihood (FIML) estimation via M*plus*. In the present real-data analysis, the factor loadings for the last two time points were freely estimated to resolve nonconvergence issues encountered in the LGM with the SI approach (see Supplementary Materials S1 and S2). The purpose of this real data analysis was to demonstrate potential discrepancies that might result when measurement errors in time-varying predictors are versus are not adjusted. The M*plus* code used in the current analyses is provided in Supplementary Materials S1 and S2.

### 2.5. Simulation Study

A simulation study was conducted to examine parameter and standard error estimate bias in the LGM with time-varying composite predictors, compared to those obtained using the LGM SI time-varying predictors. The particular parameters of primary interest are the occasion-specific effect (γ_t_) (see Equations (4) and (5)), and four design factors were varied: (1) sample size; (2) the internal consistency reliability of the time-varying predictor; (3) the number of measurement occasions; and (4) the magnitude of the time-varying predictors’ occasion-specific effects (see Table 1). A total of 1000 replication datasets were generated for each condition and estimated by using the two models (i.e., LGM composite model, LGM SI model).

#### 2.5.1. Simulation Conditions

Sample size: Conventionally, the minimum recommended sample size in the latent variable framework is five to ten observations per parameter estimated (Bentler & Chou, 1987). The number of parameters requiring estimation is equal to 21 in the LGM with SI predictors investigated here, resulting in a minimum recommended sample size of *N* = 105 when the model has four repeated measures per individual. Prior studies have noted that sample sizes of 200, 500, 1000, and 2000 are frequently seen in empirical social and behavioral science studies (Cheong, 2011; Koo et al., 2015). Thus, in alignment with simulation conditions in previous research, three different sample sizes (*N* = 100, 300, and 500) were used in generating data to represent minimum, relatively small, and moderate sample sizes, respectively; to align with scenarios in which use of the SI factor approach makes more sense than use of a fuller latent variable model for handling measurement error.

Reliability: In social science research, a reliability of 0.7 or greater is “acceptable,” 0.8 or greater is “good,” and 0.9 or greater is “excellent” in practice (Cho, 2024a, 2024b). Some researchers argue that a Cronbach’s α of 0.8 or greater is a reasonable cutoff (Gliem & Gliem, 2003). Correspondingly, the magnitude of the reliability in the observed composite time-varying predictor was varied across 0.7, 0.8, and 0.9 in this current study.

The number of measurement occasions: In modeling change over time, the number of measurement occasions has been shown to be an important factor that is related to the precision of parameter estimates in LGM (Duncan et al., 2002). The minimum required number of measurement occasions for LGM is three repeated measures, and it is typically desirable to obtain four or more measurement occasions (Fan & Fan, 2005). Hence, data were simulated with five and ten measurement occasions in the present study.

The magnitude of the occasion-specific effect coefficient: The magnitude of the occasion-specific coefficient was varied to include the following values: baseline (0.0), small effect (0.2), and moderate effect (0.5) that are typically seen in practice.

#### 2.5.2. Data Generation and Data Analysis

Data were generated according to the LGM SI model using the Monte Carlo feature in M*plus* (version 7.4; L. Muthén & Muthén, 2012). The functional form of the growth trajectory was a linear trajectory and the measurement occasions were assumed to be equally spaced. The means of the intercept and slope factors were generated to have values of zero and 0.1, respectively (Koo et al., 2015; Thoemmes et al., 2010). The disturbance variances of the intercept and slope factors were generated with respective values of 1 and 0.2 because the ratio between the disturbance variances of the intercept and slope factors is commonly reported to be 5 to 1 in practice (L. K. Muthén & Muthén, 2002). The correlations between the intercept and slope factors were set to 0.2, as commonly seen in applied research. Additionally, the means of the residuals associated with the outcome variables repeatedly measured were set to zero. The residual variances of the outcome variables were all set to equal 0.50, as in the study conducted by L. K. Muthén and Muthén (2002). These residual variances were also uncorrelated with each other across measurement occasions and with the latent growth factors’ disturbances. Future research could explore autocorrelated disturbances.

For the SI measurement model for a time-varying predictor, the factor loading of the SI factor was set to 1 and the variance of the composite time-varying predictor was set to 1. The time-varying predictor’s variance was fixed to 1 as the predictor was assumed to be a standardized score. As such, the fixed error variance of the composite time-varying predictor was set to equal [(1 − reliability) × 1] (see Equation (2)).

Once 1000 datasets were randomly generated for each condition, data were analyzed by LGM composite model and LGM SI model using M*plus* with a maximum likelihood (ML) estimation method. Subsequently, the occasion-specific effect coefficients’ parameter and standard error estimates in the two models were estimated and the robustness of parameter estimates and standard error estimates from the two models were compared using the relative parameter bias and relative standard error bias. The relative parameter bias is expressed as follows (Hoogland & Boomsma, 1998):

(6) $$B\left({\hat{\theta }}_{i}\right)=\frac{{\bar{\hat{\theta }}}_{i}-\theta_{i}}{\theta_{i}},$$

where ${\bar{\hat{\theta }}}_{i}$ is the mean of the estimates for a parameter *i* across 1000 replications, band $\theta_{i}$ is the population value of the *i*th parameter. Typically, |$\;B\left({\hat{\theta }}_{i}\right)$| of 0.05 or less is acceptable (Hoogland & Boomsma, 1998).

Additionally, the relative standard error bias was also considered in this current study. The relative standard error bias is expressed as follows:

(7) $$B\left({\hat{se}}_{{\hat{\theta }}_{i}}\right)=\;\frac{{\bar{\hat{se}}}_{{\hat{\theta }}_{i}}-\;{\hat{se}}_{\theta_{i}}}{{\hat{se}}_{\theta_{i}}},$$

where ${\hat{se}}_{\theta_{i}}$ is the estimated standard error in the population, which can be obtained by the standard deviation of the associated parameter, and ${\bar{\hat{se}}}_{{\hat{\theta }}_{i}}$ is the mean of the estimated standard errors across 1000 replications. A |$B\left({\hat{se}}_{{\hat{\theta }}_{i}}\right)$| of 0.10 or less was considered acceptable bias (Hoogland & Boomsma, 1998).

## 3. Results

### 3.1. Real Data Analysis Results

Table 2 provides comparisons of model results of the conditional LGM with and without correcting for measurement error in observed reading ability (theta) scores at each of the five time points. As seen in Table 2, the parameter and standard error estimates from the LGM composite model and the LGM SI model were different with respect to five occasion-specific effect coefficients (i.e., γ_1_, γ_2_, γ_3_, γ_4_, γ_5_ as seen in Figure 3). The parameter estimates of the five occasion-specific effect coefficients from the LGM SI model (i.e., γ_1_ = 0.709, γ_2_ = 0.578, γ_3_ = 0.518, γ_4_ = 0.701, γ_5_ = 0.739) were consistently larger than those from the LGM composite model (i.e., γ_1_ = 0.558, γ_2_ = 0.450, γ_3_ = 0.383, γ_4_ = 0.515, γ_5_ = 0.530), as expected. However, standard error estimates from the LGM SI model were marginally larger than those from the LGM composite model. With regard to the latent growth parameters, the two models provided the same magnitude of parameter and standard error estimates except for the disturbance variance of the intercept factor ($\sigma_{\zeta_{\alpha }}^{2}$). The disturbance variance of the intercept factor for the LGM composite model was 0.094, whereas that of the LGM SI model was 0.079. The model fit indices in Table 3 showed that the two models were adequately fitted to the dataset.

### 3.2. Simulation Study Results

A total of 1000 successful replications were obtained in each of the simulation conditions. When nonconvergent cases occurred, those replications were automatically rerun and replaced with converged solutions to ensure a total of 1000 valid replications. As seen in Table 4 and Table 5, for those cases with zero occasion-specific effects with five measurement occasions, the PB and RSEB results for the LGM composite model were comparable with those of the LGM SI model. Specifically, the LGM composite model’s PB and RSEB values ranged from −0.006 to 0.004 and from −0.056 to 0.035, respectively. Additionally, the LGM SI model’s PB and RSEB values ranged from −0.007 to 0.004 and from −0.069 to 0.011, respectively. Overall, the RSEB values obtained from the two models were less than 0.10, indicating acceptable RSEBs.

However, in Table 6, substantial discrepancies emerged between the RPB values of the LGM composite model and the LGM SI model. The LGM SI model’s RPB values ranged from −0.023 to 0.050 across conditions, indicating negligible bias, except for the condition with *N* = 100 and reliability of 0.7, where the RPB was slightly higher (0.052) but still minimal. In contrast, the RPB values collected from the LGM composite model ranged from −0.318 to −0.090. This means that the parameter estimates of the occasion-specific effect from the LGM composite model were substantially underestimated by 9% to 31.8%. Additionally, the RSEB values from the two models were comparable, and both of the two models’ RSEB values (see Table 7) fell within the acceptable range based on Hoogland and Boomsma’s (1998) criteria; that is, the RSEB values from the LGM SI model ranged from −0.066 to 0.035 and the RSEB values from the LGM composite model ranged from −0.058 to 0.032.

Again, when the occasion-specific effect was zero with ten measurement occasions, the LGM composite and LGM SI models provided comparable PB and RSEB values. The PB values were negligible for both models, with the composite model ranging from −0.004 to 0.004 and the SI model ranging from −0.006 to 0.006. The RSEB values were comparable with the composite model ranging from −0.059 to 0.072 and the SI model ranging from −0.086 to 0.064. In all cases, the absolute RSEB values were below 0.10, indicating acceptable standard error bias.

When the occasion-specific effect was non-zero, the performance of the two models remarkably differed in terms of parameter bias. The LGM SI model continued to show negligible bias across conditions (RPB: −0.028 to 0.050), except for a modest positive bias (RPB = 0.084) when *N* = 100 and reliability = 0.8. However, the LGM composite model exhibited substantial negative bias (RPB: −0.319 to −0.073), indicating underestimation of the occasion-specific effect by 7.3% to 31.9%. Notably, despite these differences in parameter bias, the RSEB values remained comparable across models and fell within the acceptable range suggested by Hoogland and Boomsma (1998) (SI model’s RSEB: −0.095 to 0.052; composite model’s RSEB: −0.063 to 0.056). Overall, both models performed similarly when the occasion-specific effect was absent, but only the LGM SI model maintained unbiased parameter recovery when the effect was present.

## 4. Discussion

The findings from the empirical data analysis demonstrated that the five occasion-specific effect coefficients were attenuated in the LGM composite model. This attenuation is due to measurement error in the reading theta scores predicting mathematics theta scores. When employing the SI factor approach, which explicitly models measurement error to correct for attenuation in the observed relationship, the observed effects were consistently larger in magnitude. This discrepancy in parameter estimates between the two modeling approaches (i.e., the LGM composite model and the LGM SI factor model) highlights the need for a methodological evaluation comparing their performance in terms of parameter bias and standard error recovery.

Additionally, the results from the simulation study revealed a clear discrepancy between the two models. Under conditions with nonzero occasion-specific effects, the LGM composite model produced substantially negatively biased estimates for parameters associated with occasion-specific effects, whereas the LGM SI factor model did not show such bias. Despite these differences in parameter recovery, neither model demonstrated meaningful standard error bias across the simulation conditions.

The current study extends previous work on the application of SI factor approaches to time-varying predictors within the LGM framework. Consistent with the assertions of Cole and Preacher (2014), the findings indicate that occasion-specific effects in conditional LGM may be attenuated when composite scores (e.g., means or sum scores) are used as predictors. Correcting for measurement error using the SI factor approach is particularly advantageous in applied research settings where item-level data are unavailable or where curve-of-factor (multiple-indicator) models fail to converge. Such nonconvergence issues are common in curve-of-factor models, especially with relatively small sample sizes (Hsiao et al., 2018).

Taken together, the findings from this study have important implications for educational, psychological, and social science research in which occasion-specific effects of time-varying predictors are often of primary interest, particularly in studies with limited sample sizes. These findings inform theoretically consequential modeling decisions that can shape substantive interpretations of within-person processes. Since the use of composite scores as exogenous predictors in LGM may lead to underestimation of path coefficients associated with occasion-specific effects, researchers are therefore encouraged to correct for potential measurement error in exogenous predictors by implementing the SI factor approach. However, a limitation of the present study is the use of Cronbach’s α as the sole reliability index. This is because α assumes tau-equivalence, which is found to be frequently violated in applied research. Future research could incorporate alternative reliability indices that relax this assumption. Future research could also extend this work by comparing parameter and standard error recovery between curve-of-factor and SI factor approaches for exogenous predictors in LGM. In addition, future studies may examine Type I error rates and statistical power associated with occasion-specific coefficients in LGM composite and SI factor models, given potential differences in standard error estimation. Furthermore, future research may include more realistic conditions such as larger sample sizes (e.g., *N* = 1000, 1500, or 2000) to enhance the applicability of these findings to large longitudinal datasets.

## 5. Conclusions

This study evaluated the consequences of ignoring measurement error in time-varying predictors within the LGM framework. This study also examined the performance of the SI factor approach for correcting attenuation in occasion-specific effects over time. The findings from this study showed that the use of composite scores as predictors in conditional LGM led to attenuation of occasion-specific effect estimates. Importantly, the SI factor approach yielded substantially less biased parameter estimates with unbiased standard error estimates. The findings from the current study emphasize the importance of modeling measurement error when examining occasion-specific changes over time. Moreover, occasion-specific effects are often central to substantive interpretations in developmental research in the fields of education and psychology.

Underestimation of these occasion-specific effects may lead to theoretically consequential misinterpretations. The SI factor approach offers a practical and computationally feasible alternative in situations where time-varying predictors are modeled using the known reliability. Explicit modeling of measurement error by employing the SI factor approach provides a theoretically grounded and practically viable strategy for handling potential measurement error in developmental research.

## Figures and Tables

**Table 1:** Simulation conditions manipulated in the present study.

Simulation Factors	Values
Sample size	100, 300, 500
Reliability	0.7, 0.8, 0.9
The number of measurement occasions	5, 10
Occasion-specific effect coefficient	0.0, 0.2, 0.5

**Table 2:** Parameter (and SE estimates) for each of the two conditional LGMs estimated from ECLS-K real data analysis.

	Analysis Method	
	LGM	SI LGM
Parameter	Estimate (*SE*)	Estimate (*SE*)
Mean Intercept, *μ_α_*	−0.900 (0.027)	−0.901 (0.036)
Mean slope, *μ_β_*	0.584 (0.022)	0.584 (0.024)
Intercept Variance, $\sigma_{\zeta_{\alpha }}^{2}$	0.094 (0.014)	0.079 (0.011)
Slope Variance, $\sigma_{\zeta_{\beta }}^{2}$	0.003 (0.001)	0.003 (0.001)
Intercept/Slope covariance, $\sigma_{\zeta_{\alpha }\zeta_{\beta }}$	−0.007 (0.003)	−0.007 (0.002)
Reading_1_ → Math_1_ effect ^a^, γ_1_	0.558 (0.040)	0.709 (0.044)
Reading_2_ → Math_2_ effect, γ_2_	0.450 (0.037)	0.578 (0.040)
Reading_3_ → Math_3_ effect, γ_3_	0.383 (0.045)	0.518 (0.049)
Reading_4_ → Math_4_ effect, γ_4_	0.515 (0.055)	0.701 (0.060)
Reading_5_ → Math_5_ effect, γ_5_	0.530 (0.059)	0.739 (0.066)

*Note.* SI LGM = LGM with the SI approach, *SE* = Standard error. ^a^ Reading*_i_* → Math*_i_* effect, γ*_t_* = Effect of time-varying predictor, Reading, on Math at measurement occasion *t*.

**Table 3:** Model fit indices for the two latent growth models estimated using ECLS-K data.

Model	χ2 (*df*)	CFI	TLI	RMSEA [90% CI]	SRMR
LGM	125.872 (8)	0.968	0.960	0.134 [0.114, 0.155]	0.045
SI LGM	19.355 (5)	0.996	0.992	0.059 [0.033, 0.088]	0.024

*Note.* SI LGM = LGM with the SI approach, LGM = latent growth model without the SI approach.

**Table 4:** Parameter bias (PB) of estimates of the null (zero) occasion-specific effect coefficient with five measurement occasions.

		Analysis Method									
		LGM					SI LGM				
*N*	*α*	γ_1_ ^a^	γ_2_	γ_3_	γ_4_	γ_5_	γ_1_	γ_2_	γ_3_	γ_4_	γ_5_
100	0.7	−0.001	0.003	0.000	−0.004	0.001	−0.002	0.004	−0.001	−0.006	0.000
	0.8	0.004	0.004	−0.002	0.000	−0.006	0.004	0.005	−0.003	0.000	−0.007
	0.9	−0.001	0.000	−0.002	0.002	−0.004	−0.001	0.000	−0.002	0.001	−0.004
300	0.7	−0.001	0.002	−0.003	−0.001	−0.003	−0.001	0.004	−0.004	−0.002	−0.004
	0.8	0.002	0.000	−0.001	0.003	0.003	0.002	0.000	−0.001	0.003	0.004
	0.9	0.000	−0.003	0.003	0.001	0.000	0.000	−0.003	0.003	0.001	0.000
500	0.7	0.000	0.001	0.000	−0.002	−0.001	0.000	0.001	0.000	−0.004	−0.002
	0.8	−0.001	0.002	0.001	0.000	0.001	−0.001	0.002	0.001	0.001	0.001
	0.9	−0.002	−0.001	0.000	−0.001	0.000	−0.002	−0.001	0.001	−0.001	0.000

*Note*. SI LGM = LGM with the SI approach, *α* = Reliability, *N* = Sample size. ^a^ γ*_i_* = Effect of time-varying predictor (occasion-specific effect coefficient) at time point *i*.

**Table 5:** Relative SE bias (RSEB) of estimates of the null (zero) occasion-specific effect coefficient for five measurement occasions.

		Analysis Method									
		LGM					SI LGM				
*N*	*α*	γ_1_ ^a^	γ_2_	γ_3_	γ_4_	γ_5_	γ_1_	γ_2_	γ_3_	γ_4_	γ_5_
100	0.7	−0.06	−0.021	−0.014	0.01	−0.049	−0.061	−0.046	−0.027	0.001	−0.068
	0.8	−0.023	−0.042	−0.015	0.02	−0.034	−0.029	−0.05	−0.021	0.011	−0.039
	0.9	−0.052	0.002	0.000	0.008	−0.021	−0.052	−0.005	−0.003	0.002	−0.03
300	0.7	0.008	−0.009	−0.004	−0.036	0.01	−0.001	0.004	−0.004	−0.002	−0.004
	0.8	0.01	−0.021	0.009	−0.034	0.014	0.002	0.000	−0.001	0.003	0.004
	0.9	−0.021	0.000	0.007	−0.049	−0.005	0.000	−0.003	0.003	0.001	0.000
500	0.7	−0.056	0.035	0.003	0.033	−0.022	0.000	0.001	0.000	−0.004	−0.002
	0.8	0.015	0.026	0.014	0.011	0.025	−0.001	0.002	0.001	0.001	0.001
	0.9	−0.029	−0.053	0.02	−0.008	0.004	−0.002	−0.001	0.001	−0.001	0.000

*Note*. SI LGM = LGM with the SI approach, *α* = Reliability, *N* = Sample size. ^a^ γ*_i_* = Effect of time-varying predictor (occasion-specific effect coefficient) at time point *i*.

**Table 6:** Relative parameter bias (RPB) of estimates of the non-zero occasion-specific occasion effect coefficients.

			Analysis Method									
			LGM					SI LGM				
*N*	*α*	γ	γ_1_ ^a^	γ_2_	γ_3_	γ_4_	γ_5_	γ_1_ ^a^	γ_2_	γ_3_	γ_4_	γ_5_
100	0.7	0.2	**−0.318**	**−0.287**	**−0.302**	**−0.317**	**−0.317**	0.001	0.049	0.022	0.009	0.007
		0.5	**−0.293**	**−0.294**	**−0.302**	**−0.285**	**−0.295**	0.032	0.035	0.024	**0.052**	0.036
	0.8	0.2	**−0.170**	**−0.195**	**−0.230**	**−0.199**	**−0.214**	0.050	0.030	−0.019	0.021	0.001
		0.5	**−0.193**	**−0.201**	**−0.194**	**−0.201**	**−0.212**	0.022	0.017	0.027	0.015	0.000
	0.9	0.2	**−0.111**	**−0.115**	**−0.119**	**−0.088**	**−0.098**	−0.002	−0.001	−0.009	0.029	0.014
		0.5	**−0.107**	**−0.096**	**−0.109**	**−0.099**	**−0.113**	0.001	0.016	0.000	0.011	−0.006
300	0.7	0.2	**−0.312**	**−0.298**	**−0.306**	**−0.296**	**−0.293**	−0.010	0.014	0.000	0.016	0.020
		0.5	**−0.293**	**−0.3**	**−0.301**	**−0.302**	**−0.299**	0.019	0.011	0.009	0.005	0.013
	0.8	0.2	**−0.222**	**−0.206**	**−0.198**	**−0.196**	**−0.19**	−0.023	−0.001	0.009	0.012	0.018
		0.5	**−0.204**	**−0.198**	**−0.201**	**−0.201**	**−0.197**	0.000	0.008	0.005	0.004	0.010
	0.9	0.2	**−0.100**	**−0.089**	**−0.105**	**−0.085**	**−0.103**	0.003	0.017	−0.001	0.021	0.000
		0.5	**−0.105**	**−0.102**	**−0.104**	**−0.104**	**−0.100**	−0.004	0.001	−0.001	−0.001	0.003
500	0.7	0.2	**−0.313**	**−0.312**	**−0.299**	**−0.298**	**−0.303**	−0.014	−0.013	0.007	0.008	0.004
		0.5	**−0.298**	**−0.296**	**−0.300**	**−0.301**	**−0.298**	0.008	0.011	0.004	0.003	0.008
	0.8	0.2	**−0.209**	**−0.193**	**−0.215**	**−0.213**	**−0.210**	−0.007	0.013	−0.015	−0.012	−0.010
		0.5	**−0.197**	**−0.203**	**−0.198**	**−0.201**	**−0.198**	0.007	0.001	0.006	0.003	0.006
	0.9	0.2	**−0.090**	**−0.096**	**−0.093**	**−0.100**	**−0.094**	0.013	0.007	0.011	0.002	0.009
		0.5	**−0.103**	**−0.099**	**−0.103**	**−0.102**	**−0.099**	−0.002	0.003	−0.002	0.000	0.003

*Note*. Boldfaced values of RPBs indicate non-acceptable bias. SI LGM = LGM with the SI approach, *α* = reliability, *N* = sample size, γ = occasion-specific effect coefficient. ^a^ γ*_i_* = effect of time-varying predictor (occasion-specific effect coefficient) at time point *i*.

**Table 7:** Relative SE bias (RSEB) of estimates of the non-zero occasion-specific occasion effect coefficients with five measurement occasions.

			Analysis Method									
			LGM					SI LGM				
*N*	*α*	γ	γ_1_ ^a^	γ_2_	γ_3_	γ_4_	γ_5_	γ_1_ ^a^	γ_2_	γ_3_	γ_4_	γ_5_
100	0.7	0.2	−0.004	0.039	0.014	−0.032	0.01	−0.018	0.019	−0.001	−0.044	0.005
		0.5	0.011	0.000	−0.012	−0.036	−0.052	−0.007	−0.006	−0.018	−0.050	−0.053
	0.8	0.2	−0.03	−0.007	−0.009	−0.033	−0.022	−0.037	−0.024	−0.016	−0.049	−0.036
		0.5	0.004	0.009	−0.013	0.005	0.021	−0.013	0.002	−0.021	0.001	0.021
	0.9	0.2	−0.049	−0.046	0.006	−0.002	−0.003	−0.007	−0.054	0.003	0.008	−0.014
		0.5	0.032	−0.032	−0.014	−0.058	−0.037	0.029	−0.038	−0.013	−0.066	−0.048
300	0.7	0.2	−0.024	−0.011	0.007	0.041	0.003	−0.028	−0.019	0.003	0.041	0.001
		0.5	−0.036	0.011	−0.025	0.03	−0.005	−0.041	0.001	−0.026	0.005	−0.015
	0.8	0.2	−0.03	0.009	−0.036	0.015	0.012	−0.033	0.005	−0.038	0.02	0.008
		0.5	0.006	−0.027	−0.004	−0.047	−0.055	−0.001	−0.029	−0.01	−0.047	−0.059
	0.9	0.2	0.004	0.002	−0.015	0.015	−0.002	0.003	0.000	−0.019	0.011	−0.005
		0.5	−0.019	0.009	−0.009	−0.02	−0.03	−0.023	0.01	−0.013	−0.021	−0.029
500	0.7	0.2	0.013	0.014	−0.006	−0.031	−0.007	0.014	0.018	−0.002	−0.034	−0.013
		0.5	−0.01	−0.013	−0.008	−0.025	−0.002	−0.005	−0.013	0.000	−0.036	−0.008
	0.8	0.2	0.008	0.008	0.023	0.014	−0.034	0.006	0.004	0.025	0.015	−0.036
		0.5	−0.007	−0.044	−0.008	−0.013	−0.006	−0.013	−0.049	0.002	−0.012	−0.01
	0.9	0.2	−0.005	−0.022	−0.019	−0.011	0.034	−0.004	−0.027	−0.022	−0.017	0.035
		0.5	−0.002	0.014	0.029	−0.013	−0.03	−0.002	0.02	0.020	−0.014	−0.025

*Note*. SI LGM = LGM with the SI approach, *α* = reliability, *N* = sample size, γ = occasion-specific effect coefficient. ^a^ γ*_i_* = effect of time-varying predictor (occasion-specific effect coefficient) at time point *i*.

## Data Availability

The data supporting the findings of this study are available from the corresponding author upon request.

## Supplementary Materials

The following supporting information can be downloaded at: https://www.mdpi.com/article/10.3390/bs16060855/s1.
