# Supplementary material for: A Single-Indicator Factor Approach for Correcting Measurement Error in Time-Varying Predictors in Developmental Research
Source: Behav Sci (Basel). 2026 May 27;16(6):855. doi: 10.3390/bs16060855 (PMC13295218; doi:10.3390/bs16060855)
Supplement: Supplementary file 1 [file behavsci-16-00855-s001.zip › behavsci-4095507-supplementary.pdf]

Supplementary Materials for "A Single-Indicator Factor Approach for Correcting Measurement Error in Time-Varying Predictors in Developmental Research"

Supplementary A

M*plus* code for latent growth modeling with a time-varying predictor

```
TITLE: REAL DATA ANALYSIS - LGM WITH COMPOSITE SCORE

DATA: FILE IS C:\Users\FINAL.CSV;

VARIABLE: NAMES ARE ID R1 M1 R2          M2       R4       M4       R5       M5
          R6       M6;
USEVARIABLES ARE M1 M2 M4 M5 M6 R1 R2 R4 R5 R6;

MODEL:

      ! THIS IS FOR RUNNING A LINEAR GROWTH MODEL
      I S | M1@0 M2@1 M4@3 M5*7 M6*11;
      I WITH S;
      ! THE LAST TWO TIMEPOINTS SHOULD BE FREELY ESTIMATED TO
ENSURE MODEL CONVERGENCE

      ! TIME-VARYING COMPOSITE PREDICTORS
      M1 ON R1;
      M2 ON R2;
      M4 ON R4;
      M5 ON R5;
      M6 ON R6;


OUTPUT: STDYX TECH1;
```

Supplementary B

M*plus* code for latent growth modeling with a single-indicator factor predictor

```
TITLE: REAL DATA ANALYSIS - LGM WITH SINGLE INDICATOR FACTOR

DATA: FILE IS C:\Users\ FINAL.CSV;

VARIABLE: NAMES ARE ID R1 M1 R2          M2      R4      M4      R5      M5
        R6      M6;
USEVARIABLES ARE M1 M2 M4 M5 M6 R1 R2 R4 R5 R6;

MODEL:

    ! THIS IS FOR RUNNING A LINEAR GROWTH MODEL
    I S | M1@0 M2@1 M4@3 M5*7 M6*11;
    I WITH S;
    ! THE LAST TWO TIMEPOINTS SHOULD BE FREELY ESTIMATED TO
ENSURE MODEL CONVERGENCE



    ! SINGLE-INDICATOR FACTOR PREDICTORS
    F1 BY R1@1;
    F2 BY R2@1;
    F4 BY R4@1;
    F5 BY R5@1;
    F6 BY R6@1;

    ! RESIDUAL VARIANCES OF THE COMPOSITE SCORES
    [R1 R2 R4 R5 R6@0];
    R1@0.026; R2@0.014; R4@0.009; R5@0.007; R6@0.008;


    ! TIME-VARYING SINGLE-INDICATOR FACTOR PREDICTORS
    M1 ON F1;
    M2 ON F2;
    M4 ON F4;
    M5 ON F5;
    M6 ON F6;


OUTPUT: STDYX TECH1;
```
